# The Future of the Hospitals

**Published:** 1921-12

**Authors:** William J. Collins


### THE FUTURE OF THE HOSPITALS.

### THE CAVE COMMITTEE AND AFTER.

By SIR WILLIAM J. COLLINS, K.C.V.O., M.S., M.D., B.Sc.(Lond.), F.R.C.S. Eng.).

TWO or three years ago there was much talk of the voluntary hospitals being taken over by the State. The anonymous medical correspondent of "The Times" informed us* that "it is becoming more and more evident that these institutions, which are the training centres of doctors, must be brought within the control of the State." The "Pall Mall Gazette" oracularly declared†: "There can be little doubt that when the Health Ministry comes into being one of the first and most important duties will be to take over the voluntary hospitals and establish them on a national basis." Dr. Addison in cryptic utterance observed (April 23, 1920): "It is inconceivable that any system of health authority could be set up which did not include the hospital system," while Lord Astor, on the termination of the Committee stage of the Ministry of Health Bill, in rounded phrase declared‡: "The whole question of hospitals was one which the Minister would have to go into. It was the intention, as soon as the Ministry of Health was established, to inquire into a large number of questions connected with the health and welfare of the community at large." A State Medical Service was freely spoken of as an advance or an improvement upon the panel service of the National Health Insurance Act.

The Ministry of Health in due time replaced the Local Government Board, and Dr. Addison's Miscellaneous Provisions Bill of 1920 contemplated, *inter alia*, further steps in the direction of municipalising hospitals. After much criticism and surrender of clauses in the Commons, this luckless measure was finally rejected in the Lords. A "hospital crisis" was declared to be imminent, and a Departmental Committee, under the chairmanship of Lord Cave, was appointed in January 1921 "to consider the present financial position of the Voluntary Hospitals, and to make recommendations as to any action which should be taken to assist them." The Committee had scarcely sat three months before they unanimously issued an interim report to the effect that "it is desirable, in the public interest, to maintain the voluntary system of hospital management." On May 31, 1921, in their final report, they found unanimously that "it would be lamentable if by our apathy or folly it (the voluntary system) were suffered to fall into ruin." No definition of what was meant by "the voluntary system" was vouchsafed by the Committee. They did, however, by their findings, indicate certain things which they held would be incompatible with its continuance. Thus they were satisfied that regular or continuous State or municipal aid would be "fatal to the voluntary system." The Committee further considered that voluntary contributions invited from patients are "more in accordance with the voluntary principle" than "a fixed charge as a condition of admission." Again, they hold that the extension of the practice obtaining in some hospitals of paying portions of grants from public authorities to the staff would "endanger the future of the voluntary hospitals."

Now, without attempting a definition of "a voluntary hospital," the expression may be said to be generally held to imply: (1) That the hospital is supported mainly by voluntary subscriptions or endowments; (2) that the permanent staff consists of honorary physicians and surgeons who render voluntary and gratuitous services to the sick and injured poor; (3) that eligible patients are freely admitted and any contributions which they may make are voluntary offerings; and (4) that the hospital is managed by an unpaid voluntary Board, elected by the subscribers and independent of bureaucratic control by State or municipal officials.

It is, however, undoubtedly true that these characteristics have been largely undermined in recent years, often with the cordial but short-sighted support of some who profess and call themselves ardent admirers of the voluntary system. Increasing subsidies have been sought and accepted from public funds, not merely by way of donations towards general maintenance, but by payment, in some cases, of the full cost of work done. Thus State money is received by the hospitals for the treatment of venereal disease, for maternity and infant welfare, for the training of nurses, and by the medical schools for education. The County County Council pays for medical treatment of school children, and Borough Councils for treatment of tuberculous diseases. In some cases, according to the Cave Report, some portion of the grants from public authorities "has been divided among the staff," while in view of these payments officials of the State and County Council have intervened in matters of administration. It has thus become a question not so much as to whether the voluntary system shall be preserved intact, as whether the undermining process shall go further or whether it can be arrested or perhaps reversed.

There are, indeed, those who have clamoured for the payment by public authorities of the "full cost, including a proper share of overhead charges, interest on capital, depreciation, etc., for all tuberculosis patients, venereal patients, maternity patients, pensioners, school children, insured persons, and other cases, if any," and also retrospectively for the full cost to the hospitals for treating our sick and wounded soldiers and sailors in the Great War. This latter demand would appear to be especially unhandsome, seeing that during the war the voluntary hospitals made the proud and just

* "Times," December 9, 1918.
† "Pall Mall Gazette," March 17, 1919.
‡ "Times," March 28, 1919.

**The Future of the Hospitals—(*cont.*).**

claim that they were shouldering the burden of the State in opening their wards to our sick and wounded heroes, although receiving, by way of "grants-in-aid," only a proportion of their actual cost of maintenance. This claim met with generous response from the benevolent public, and it would ill become the hospitals at this date to submit a bill of full retrospective payment for a work which they gladly undertook and nobly discharged. The Cave Committee happily appears to have had no sympathy with this unfortunate suggestion.

Another scheme for assisting the voluntary hospitals, of the nature of Provident Insurance, was also pressed upon the notice of the Cave Committee, commonly called the "Sussex scheme," although it does not appear to have been generally adopted in that county. By it a subscription of £1 to £2 per annum, according to income limit (which in no case should exceed £500 per annum per family), guarantees free consultation and treatment in hospital (or at own home at reduced fees), with *x*-ray, electrical, and massage treatment, laboratory investigation, and district nursing. As the Cave Committee remarks, it is "not yet fully established"; three or four London hospitals have, however, since taken it up, sometimes without the concurrence of their staff. The Committee reports that "the main objection to the scheme is that it implies a contract on the part of the hospitals" which it may be impracticable to fulfil. Other not less serious objections which have been raised are that it fundamentally alters the object and purpose of the voluntary hospital, and, instead of pursuing the Good Samaritan work of ministering to the sick poor, tends to convert hospitals into subsidised nursing homes for the benefit of a better-off class, thus risking the alienation of the philanthropic public and the honorary visiting staff.

Then there is the problem of the hospital treatment of insured persons. When the National Health Insurance Act was passed in 1911, with its loud-mouthed preamble to the effect that it was "to secure adequate medical attendance and treatment for all insured persons, including drugs, medicines, and appliances," many proclaimed that Othello's occupation was gone and that the knell of the voluntary hospitals had been sounded. But it transpired that in the process of translating this magniloquent preamble into action, the Commissioners discovered that all that was meant was "only such services as are ordinarily rendered by a general practitioner to his patients in private practice." The hospitals lost subscribers, but they did not lose their in-patients. The curious paradox was thus brought to pass—namely, that if a disease or accident were relatively not very serious, it was an affair for the State service; if, however, a disease was grave or an accident a severe one, it was a case for the voluntary hospitals. Accordingly insured persons found their way in large numbers into the wards of the voluntary hospitals; in some cases 50 per cent. of the inmates of male wards were, and are, found to be insured persons.

While the Insurance Act made it lawful for an Approved Society or an Insurance Committee to contribute to voluntary hospitals, few, if any, availed themselves of the privilege. A great demand was made in some quarters that the hospitals should be paid the full cost of treating insured persons, and the huge surplus funds of the Approved Societies disclosed by the quinquennial valuation were pointed to as a legitimate source for financial assistance to the insolvent hospitals.

The Cave Committee in its interim report of March 9, 1921, estimated this "disposable surplus" at £7,000,000, and was strongly of opinion that the schemes for additional benefits about to be submitted to the Minister of Health by the Approved Societies should provide for substantial subventions to hospitals treating their members, and that approval to such schemes should be withheld pending due consideration of this recommendation. In its final report, however, the Cave Committee struck a different note; it held that the suggestion that the cost of maintenance of insured persons in a voluntary hospital should fall on insurance funds rested upon a misconception, and that no discrimination should be exercised against insured persons. The Approved Societies, it added, are not under any obligation to provide the cost of hospital treatment of their members, but the Committee hoped that the "generous example" of one Society in devoting a portion of its surplus for the benefit of hospitals might be followed by others.

As is well known, the chief final recommendations of the Cave Committee were that Parliament should make a temporary grant of £1,000,000 to help the voluntary hospitals to tide over the "crisis," that a Hospitals Commission should be set up for two years to administer this grant *plus* a sum of £250,000 for extensions and improvements, and that local voluntary hospital committees should be formed, with no compulsory powers, but with a view to promote co-operation among hospitals within the local area, to systematise appeals and prevent competition and overlapping.

The Minister of Health, in nominating the Hospitals Commission, with Lord Onslow as Chairman, announced that the Government would not contribute towards building schemes and would limit their grant to £500,000, which is to be so administered "as to secure that the necessity for such a grant shall disappear as quickly as possible." It now transpires that only a portion of the half-million grant is to be distributed this year, and such distribution has been further conditioned by requiring the hospitals to raise an equivalent amount of "fresh money" by their own efforts, a condition whose equitable application will prove exceedingly difficult. Meanwhile a "Policy Committee" of the King's Hospital Fund has been conferring with the Metropolitan hospitals and hospital collecting agencies in London with a view to securing larger contributions from wage-earners, either by intensifying present efforts or by new methods. One of

**The Future of the Hospitals—(*cont.*).**

the latter contemplates dissecting London up into "hospital areas ranging round key hospitals," and one or two rather costly schemes have been adumbrated for this purpose. It must, however, be borne in mind that this "key hospital scheme," the "National Provident Scheme," and the new practice of urging or requiring payment by in-patients are to some extent incompatible the one with another, and the "industrial classes" against whom they are all more especially directed will not be slow to detect such incompatibility and possibly to resent it.

While, therefore, it is good to know that "the Government accept the view of the Cave Committee that the Voluntary System must be maintained," their asseveration that "they cannot admit that it is incumbent on the Government to find money to make up the present deficit on the voluntary hospitals" leaves the problem of financing the hospitals very much as it was before, only a little more so.

